# Adult psychiatrists’ views on clozapine prescribing for schizophrenia in Germany—an online survey

**DOI:** 10.1177/20451253261434380

**Published:** 2026-04-17

**Authors:** Mishal Qubad, Ida Marie Ehret, Christian J. Bachmann, Robert A. Bittner

**Affiliations:** Goethe University Frankfurt, University Hospital, Department of Psychiatry, Psychosomatic Medicine, and Psychotherapy, Heinrich-Hoffmann-Str. 10, Frankfurt am Main 60528, Germany; Ernst Strüngmann Institute of the Max Planck Society, Frankfurt am Main, Germany; Goethe University Frankfurt, University Hospital, Department of Psychiatry, Psychosomatic Medicine, and Psychotherapy, Frankfurt am Main, Germany; Department of Child and Adolescent Psychiatry, Ulm University, Ulm, Germany; Goethe University Frankfurt, University Hospital, Department of Psychiatry, Psychosomatic Medicine, and Psychotherapy, Frankfurt am Main, Germany; Ernst Strüngmann Institute of the Max Planck Society, Frankfurt am Main, Germany

**Keywords:** antipsychotics, attitude, clozapine, treatment-resistant schizophrenia

## Abstract

**Background:**

Clozapine remains the only effective antipsychotic drug for treatment-resistant schizophrenia (TRS), yet it continues to be markedly underused in most industrialized countries including Germany. Previous studies have identified prescriber-related factors such as concerns about adverse drug reactions, the burden of mandatory monitoring, and limited experience with clozapine use and TRS recognition as major contributors to this persistent underutilization. However, these issues have not been studied for the German healthcare system.

**Objectives:**

To investigate prescriber attitudes toward clozapine use for schizophrenia in Germany and identify related treatment barriers.

**Design:**

Cross-sectional, web-based survey study.

**Methods:**

We conducted a web-based cross-sectional survey using PsyToolkit. Our questionnaire assessed clinicians’ demographics, familiarity with relevant national guidelines, practical experience with clozapine and formalized training in its use, perceived treatment barriers, and presumptions about patients’ attitudes toward clozapine. Data were predominantly analyzed descriptively.

**Results:**

A total of 155 psychiatrists—most of them board-certified and nearly all regular clozapine prescribers for schizophrenia—completed the survey. Most participants were familiar with guideline recommendations for clozapine initiation. However, even among them, most preferred to attempt at least one trial of antipsychotic polypharmacy before starting clozapine. Formalized training had a positive impact on knowledge regarding clozapine’s effectiveness in reducing negative symptoms, aggressive behavior, and suicidality. While most participants acknowledged clozapine’s effectiveness in reducing all-cause mortality, only a small proportion of participants acknowledged its effectiveness in reducing cardiovascular mortality. Notably, three quarters of participants presumed that patients would prefer standard antipsychotics over clozapine. Monitoring requirements and concerns regarding weight gain and blood dyscrasia were ranked as the main barriers impeding clozapine use.

**Conclusion:**

We identified several modifiable prescriber-related factors limiting clozapine use for schizophrenia in Germany. Implementing mandatory targeted training programs during residency and regular use of shared decision-making to emphasize the patients’ perspective might facilitate a timelier and widespread use of clozapine.

## Introduction

### Clozapine for treatment-resistant schizophrenia

Thirty percent of all individuals with schizophrenia develop a treatment-resistant course (treatment-resistant schizophrenia, TRS).^1–7^ Notably, 80% of these cases emerge during the first psychotic episode.^6,8–10^ TRS is associated with a disproportionately high load of stigma, illness burden, and healthcare costs for affected patients, underscoring the need for adequate evidence-based treatment. To date, clozapine remains the only effective antipsychotic for TRS.^11–15^ This is the most up-to date meta-analysis demonstrating clozapine’s unique efficacy for TRS.^16^ Clozapine entails the lowest risk for extrapyramidal adverse drug reactions (ADRs).^17^ Moreover, meta-analytical and real-world data have repeatedly demonstrated clozapine’s clinical superiority compared to standard antipsychotics.^17–27^ Specifically, clozapine is superior in improving global psychopathology, positive symptoms, and negative symptoms. Furthermore, clozapine has been shown to be superior in increasing treatment adherence, in reducing aggressive behavior as well as relapse risk in comorbid substance-use disorder.^12,17,18,20–22,28^ Importantly, in addition to its anti-depressant effects, clozapine minimizes suicidal behavior.^12,17,25,29–32^

Moreover, despite its metabolic ADRs, clozapine does reduce cardiovascular mortality.^19,33–36^ Most importantly, compared to standard antipsychotics, clozapine is superior in decreasing all-cause and suicide mortality.^19,27,37^ Its effects on all-cause mortality have been partially attributed to its superiority in increasing treatment adherence for somatic comorbidities.^26^ Notably, a recent population-based cohort study reported that compared to standard antipsychotics including long-acting injectables, clozapine significantly reduces relapse risk in first-episode patients, suggesting that it may already be appropriate and beneficial as a second-line treatment.^38^ Overall, given these unique benefits, current consensus guidelines recommend clozapine as a second-line treatment for patients with persistent positive symptoms accompanied by suicidality, aggression, or ADRs such as extrapyramidal symptoms or tardive dyskinesia.^39^

For TRS, response rates to clozapine range from 40% to 60%.^25,38,40–44^ Notably, mirroring findings for the delay of untreated psychosis, a delay in clozapine initiation decreases response rates and has a clear detrimental impact on long-term outcome.^40,42,45,46^ Therefore, timely initiation of clozapine using therapeutic drug monitoring is essential for maximizing patients’ benefits.^40,42,45^

### Additional clozapine indications

Importantly, meta-analytic and register-based evidence also indicates broad beneficial effects in bipolar disorder,^37,47,48^ major depressive disorder, and psychotic depression.^49^ Additionally, clozapine’s anti-aggressive effects appear to extend to bipolar disorder, post-traumatic stress disorder, borderline personality disorder, autism spectrum disorder, and learning disability.^50,51^ Moreover, clozapine also shows clear efficacy in psychosis associated with Parkinson’s disease.^37,52–56^ This underscores the broad relevance of clozapine underprescribing.

### Clozapine underutilization

National and global clinical guidelines recommend initiating clozapine after two unsuccessful trials of standard antipsychotics at adequate doses and durations,^57,58^ in line with the definition of TRS.^5,58^ Despite these clear recommendations, clozapine remains underutilized across industrialized countries,^59^ in favor of less effective strategies, including antipsychotic polypharmacy, antipsychotic dosing exceeding recommended levels, and augmentation with mood stabilizers.^5,40,60^

In Germany, clozapine prescription rates are estimated at 95 per 100,000, whereas the prevalence of TRS is about 200 per 100,000 individuals, clearly indicating that current prescribing remains insufficient.^59^ Low clozapine prescription rates have also been observed in most other industrialized countries, except for Finland, where intensive national efforts have resulted in a substantial increase of clozapine prescription rates over recent decades.^40,59^

### Factors limiting clozapine use

The causes underlying clozapine underutilization have been studied in both industrialized and developing countries.^61–85^ There is converging evidence that patients’ attitudes and treatment decisions are not a major issue.^40,86–88^ Furthermore, prescribers often anticipate patient reluctance toward clozapine, primarily due to monitoring requirements and concerns about ADRs. They also assume lower patient satisfaction with clozapine, which increases the likelihood that it is not even offered as a treatment option.^40,89^ However, the majority of patients clearly prefer clozapine over their previous antipsychotics despite the associated monitoring requirements and potential ADRs.^81,86,87,90^

Notably, clozapine underutilization appears to be primarily due to prescriber-related factors. Converging evidence indicates that prescribers perceive mandatory monitoring requirements as a significant burden.^61–85^

Moreover, the majority of prescribers report concerns about clozapine-associated ADRs, particularly clozapine-induced neutropenia (CIN)^91^ and agranulocytosis (CIA), and tend to overestimate the risks of these ADRs.^92–95^

Limited experience with clozapine use and ADR management have also emerged as a barrier.^61–70,72–85^ Interestingly, in a recent Danish survey, psychiatrists considered some patients to be either too ill or too well-treated to receive clozapine, even when symptom severity remained high.^70^ Moreover, psychiatrists tend to apply more restrictive eligibility, for example by requiring the presence of subjective distress from persistent symptoms in addition to high symptom severity.

Country-specific barriers include mandatory patient registration,^75,85^ healthcare professional certification, in-patient clozapine titration during the first eighteen weeks, established collaboration with cardiologists, hematologists, and diabetologists, as well as unnecessarily strict hematological thresholds.^66,72,75^ Finally, in many developing countries, limited access to healthcare is a key factor limiting clozapine use.^78^ For Germany, prescriber-related factors contributing to the underutilization of clozapine have not been systematically studied. Given differences in healthcare systems, factors identified in other industrialized countries may not be directly applicable to Germany.

### Mental healthcare in Germany

Compared with other European countries, Germany allocates a considerable proportion of its Gross Domestic Product to healthcare, including mental healthcare.^96–98^ Health insurance has been compulsory since 2009 and is organized as a dual system.^96,99,100^ Approximately 89% of the population is covered by non-profit statutory health insurance.^96,99,101^ About 11% of the population is insured through private schemes.^96,99,101^ Despite compulsory coverage, 0.1% of the total population remains uninsured.^96,99,100^ For these individuals, access to emergency but not long-term medical care is covered by the social welfare system.^96,102,103^

Mental healthcare is primarily delivered through hospitals and private practices and is generally covered by health insurances.^104^ Hospitals typically provide inpatient, outpatient, and emergency care, whereas private practices, run by board-certified psychiatrists, offer outpatient services only.^104^ Among European countries, Germany has the highest proportion of psychiatric hospital beds, which are consistently occupied near full capacity.^105–107^ In addition, psychiatric day hospitals are widely established across the country,^104^ while multi-disciplinary home treatment services remain scarce.^96,108^

The fragmented structure of the German mental healthcare system represents a substantial barrier to timely access to treatment.^108^ Compared with the United Kingdom, Finland, Denmark, and Australia, Germany has a markedly lower availability of Youth Mental Health Services and early intervention services (EIS) for individuals experiencing first-episode psychosis (FEP),^96,109–114^ resulting in a longer duration of untreated psychosis.^113,115,116^

Typically, FEP patients first seek care from mental health professionals in private practices or hospitals and ideally remain in treatment within these settings over the course of their illness.^116–119^ In contrast, general practitioners are less likely and less well trained to provide care for people with severe mental disorders.^96,119^ Furthermore, dedicated “clozapine clinics,” that is, services specialized in clozapine initiation and management, are not well established in Germany.^40^

### Psychiatry residency training in Germany

Psychiatry residency training in Germany is regulated by state-level curricula issued by the 16 state-branches of the National Board of Medical Professionals.^120,121^ Training has a minimum duration of 5 years and includes 24 months of mandatory inpatient psychiatry and 12 months of neurology. The remaining 24 months may be completed in general psychiatry in inpatient or outpatient settings or may include up to 12 months in forensic psychiatry, child and adolescent psychiatry, or psychosomatic medicine^120^ Notably, up to six of these 12 months may be completed in neuropathology, neurosurgery, or internal medicine.^120^

Typically, residents must fulfill a catalogue of minimum training requirements, including a specified number of supervised psychotherapy sessions, and pass an oral clinical examination to obtain board certification.^122–124^ Importantly, none of the state-level curricula mandate specific psychopharmacological training, including training in clozapine use.^121,125^

Psychiatric hospitals constitute the primary accredited training sites for psychiatry residency in Germany.^126–128^ These include psychiatric departments within university hospitals, psychiatric units in general hospitals, and stand-alone psychiatric hospitals. In addition, residents may complete up to 24 months of their training in certified private practices run by board-certified psychiatrists.^129,130^

### Hypotheses

We aimed to assess prescriber-related factors contributing to clozapine underutilization in Germany using an online survey. Based on the current state of research, we hypothesized to find low clinician familiarity with and adherence to guidelines for clozapine initiation and prescribing. Moreover, we expected a preference for polypharmacy and substantial concerns regarding ADRs, particularly blood dyscrasia. We also posited that prescribers would regard monitoring requirements as a burden impeding clozapine use and that they falsely assume that patients would prefer standard antipsychotics over clozapine. Finally, we hypothesized that formalized training in addition to supervised use of clozapine during residency increases both knowledge regarding clozapine’s effectiveness and the rate of guideline-based prescription behavior.

## Methods

We conducted a cross-sectional study using a web-based survey in Germany from August 2024 to July 2025. The reporting of this study adheres to the “Strengthening the Reporting of Observational Studies in Epidemiology (STROBE)” guidelines (Supplemental File 1).^131^

### Survey development and topics

The survey was developed utilizing the web-based platform PsyToolkit 3.4.6 and consisted of 38 items (Supplemental File 2).^132,133^ The duration of the survey was approximately 15–20 min. The survey targeted residents and board-certified psychiatrists. The questionnaire was partly adapted from a previous study with permission from the authors.^77^ The questionnaire was not formally validated but piloted in three late-stage psychiatric residents.

We collected information on the participants’ age, sex, professional demographics including level of post-graduate education (resident vs board-certified) and experience, current workplace, formalized training and knowledge related to clozapine prescription and ADR management, as well as clozapine prescription patterns (Table 1, Supplemental File 2).

**Table 1.** Major topics covered with the survey.

**General information/demographics**
Participants age distribution
Level of post-graduate education
**Current workplace**
Training type in clozapine use: e.g., supervised use in clinical setting and/or formalized training
Case load: number of patients with schizophrenia and number of patients treated with clozapine under the participants’ care
**Knowledge**
Current national guideline recommendations on when to start clozapine
Clozapine effectiveness regarding negative symptoms, suicidal behavior, aggressive behavior, all-cause mortality, cardiovascular mortality
**Perceived barriers**
Adverse drug reactions perceived as barriers
Management/monitoring requirements perceived as barriers
Perceived barriers limiting clozapine use from the patient perspective
Assumptions regarding patients’ medication preferences

For investigating the impact of formalized training, we distinguished between supervised use during residency under a consultant psychiatrist, formalized training independent of the previously mentioned supervised use, a combination of both, and no training at all. Our survey also included questions about the number of patients with schizophrenia-spectrum disorder under the participant’s care, as well as questions about the number of patients treated with clozapine under the participant’s care.

For specifying participants’ workplace, we primarily distinguished between three categories: psychiatric departments at university hospitals, other psychiatric hospitals, that is, psychiatric departments within general hospitals and stand-alone psychiatric hospitals, and private practices.

Furthermore, we collected information on the participants’ knowledge regarding clozapine-related recommendations provided by the national guidelines,^59,134^ as well as the participants’ attitudes toward clozapine and polypharmacy with standard antipsychotics.

Participants were also asked to rate clozapine’s effectiveness in reducing negative symptoms, aggressive behavior, suicidality, all-cause mortality, and cardiovascular mortality using a four-point Likert-type scale (“Yes”, “Likely Yes”, “Likely No”, “No”). For descriptive purposes, responses were dichotomized into agreement (“Yes”, “Likely Yes”) and disagreement (“Likely No”, “No”). Using a similar four-point scale, participants further evaluated patients’ presumed treatment preferences by indicating whether they believed patients would prefer clozapine (“Yes”, “Likely Yes”) or standard antipsychotics (“Likely No”, “No”).

Furthermore, we collected information on perceived barriers limiting the widespread use of clozapine. In this context, we distinguished general barriers attributed to clozapine use—such as mandatory monitoring requirements and slow titration—from barriers related to clozapine associated ADRs. With respect to general barriers, participants had to rank six potential barriers in order of significance, with one indicating the most significant and six the least significant. With respect to ADRs perceived as barriers to long-term use, participants ranked each from one, indicating the most important obstacle, to nine, indicating no obstacle. Finally, we asked participants to rank patient-related barriers to clozapine use in the same way. Participants ranked each from one, indicating the most important obstacle, to five, indicating the least important obstacle.

### Survey distribution

To maximize the reach of our survey, we contacted multiple national networks for clinical psychiatry with the aim of engaging an unbiased sample of psychiatrists working in both inpatient and outpatient settings throughout Germany. To this end, we contacted every professional network for German psychiatrists. For psychiatrists working in hospital settings, these networks included “Lehrstuhlinhaber für Psychiatrie und Psychotherapie (LIPPs e.V.)”, “Bundesdirektorenkonferenz (BDK)”, and “Arbeitskreis der Chefärztlnnen der Kliniken für Psychiatrie und Psychotherapie an Allgemeinkrankenhäusern in Deutschland (ACPA)”. For psychiatrists working in outpatient settings, that is, in private practices, this included “Berufsverband Deutscher Nervenärzte (BVDN e.V.)”. For organizations representing psychiatrists working in either setting, we contacted the “German Association for Psychiatry, Psychotherapy and Psychosomatic Medicine (DGPPN e.V.)”. The survey invitation, including the survey URL, was distributed by email.

### Sample size calculation

The study was designed as an exploratory, cross-sectional survey of psychiatrists working in inpatient and outpatient settings across Germany. Due to the anonymous nature of the survey and the distribution via national professional networks without access to a complete sampling frame or response denominator, an a-priori sample size calculation or formal power analysis was not feasible. Consequently, no predefined target sample size was specified. Instead, recruitment was guided by feasibility and comparability with prior national-level surveys conducted in other countries investigating prescriber attitudes toward clozapine, which typically report sample sizes ranging from approximately 80 to 280 participants.^66,68,76,79,135^ Within this context, the final sample of 155 completed questionnaires was considered sufficient to allow robust descriptive analyses and exploratory group comparisons, particularly regarding the impact of training exposure on prescribing attitudes and clozapine-related knowledge.

### Statistics

We used descriptive statistics to summarize the participants’ answers. To assess the impact of training type in clozapine management, that is, supervised clozapine use versus supervised clozapine use and additional formalized training, on polypharmacy preference, we applied Pearson Chi-squared tests.

To assess the impact of training type on participants’ assumptions whether patients preferred clozapine over standard antipsychotics, responses were first dichotomized into “agreement” (“Yes” and “Likely Yes”) and “disagreement” (“No” and “Likely No”) for descriptive purposes. Moreover, to account for the original nature of the four-point-Likert-type responses, we conducted a Mann–Whitney *U* test to assess the impact of training type on the participants’ assumptions regarding patients’ preferences. Likert-type items were treated as ordinal variables. Accordingly, results are reported using medians and interquartile ranges, and group comparisons were performed using non-parametric tests. No parametric statistics were applied to single-item Likert-scale data.

To assess the impact of training type on knowledge regarding clozapine effectiveness, we also conducted a Mann–Whitney *U* test. Statistical analyses were performed using SPSS version 31.^136^

## Results

### Demographics

Because the survey was conducted anonymously online and recruitment relied on the previously mentioned professional networks, it was not possible to determine how many board-certified psychiatrists and residents received the invitation. According to the analytics of our website, a total of 403 people accessed the survey page, of whom 21 started but did not complete the survey, and 155 individuals completed it. Importantly, we cannot determine whether incomplete survey entries and page visits were generated by individuals who later completed the survey or accessed it from another device. Therefore, all analyses are based on these 155 individuals.

Details regarding professional demographics are depicted in Table 2. The analysis of age distribution revealed a predominance of middle-aged and older prescribers, 41% of participants were females (Table 2). Most participants reported having more than 30 years of experience in medicine (Table 2). Ten percent of participants were residents, whereas 90% of participants were board-certified psychiatrists. Sixteen percent of participants were employed at psychiatric departments of university hospitals, 55% of participants were employed at other psychiatric hospitals, and 23% of participants were employed in private practices. The remaining 6% of participants were either retired or currently employed in rehabilitation centers, neurological departments, or private practices specializing in neurological disorders.

**Table 2.** Demographic characteristics of participants.

Participant characteristics	Sample (*n* = 155)
**Sex (%)**	
Females	41
Males	59
**Age distribution [%]**	
20–40 years	23
41–60 years	52
>60 years	25
**Level of post-graduate education (%)**	
Residents	10
Board-certified psychiatrists	90
**Years of experience in medicine (%)**	
1–10 years	20
11–20 years	27
21–30 years	25
>30 years	28
**Workplace (%)**	
Psychiatric departments at university hospitals	16
Other psychiatric hospitals	55
Private practices	23
Other	6
**Training types in clozapine use (%)**	
Supervised use during residency only	49
Supervised use during residency and additional formalized training	41
Formalized training only	3
None	7

### Training in clozapine use

A total of 49% of participants reported having learned clozapine management only through supervised use with their consultant psychiatrist during residency (Table 2). Three percent of participants reported having learned clozapine management only through formalized training outside the hospital they completed their residency training in. A total of 41% of participants reported having learned clozapine management through both means, that is, under the supervision of their consultant psychiatrist during residency as well as through additional formalized training. Seven percent of participants (*n* = 11) reported never having had any training in clozapine management.

### Professional experience

**Figure 1.**
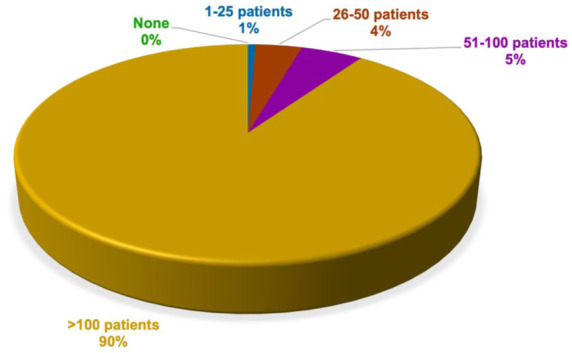
Caseload of patients with schizophrenia during career. All participants working in psychiatric hospitals and private practice reported having acquired experience in treating patients with schizophrenia during their career and currently treating patients with schizophrenia. The vast majority of participants reported having treated more than 100 patients with schizophrenia over the course of their career.

All participants reported having acquired experience in treating patients with schizophrenia during their career. The distribution of the number of people diagnosed with schizophrenia during career is shown in Figure 1.

**Figure 2.**
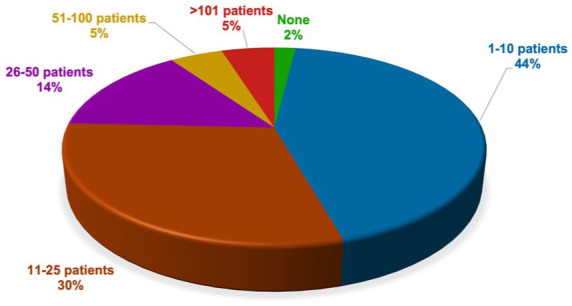
Current caseload of patients with clozapine in outpatient settings. Among all participants, the majority reported to currently treating patients with clozapine in outpatient settings. The majority of participants reported currently treating either 1–10 or 11–25 patients with clozapine in outpatient settings.

**Figure 3.**
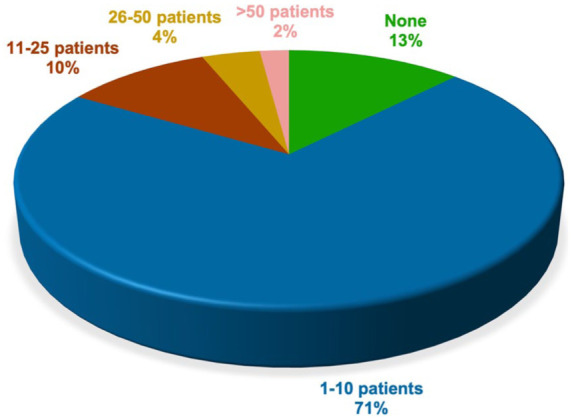
Current caseload of patients on clozapine in inpatient settings. Among all participants, the majority reported currently having patients on clozapine under their care. A clear majority of participants reported currently treating 1–10 patients with clozapine in inpatient settings.

Almost all participants (*n* = 153) reported having acquired experience in treating patients with clozapine during their career. The remaining two were residents in their first and fifth years of training, respectively. A total of 88% of participants reported that they were currently having patients treated with clozapine under their care. The highest proportion of patients currently treated with clozapine by the participants fell within the range of 1–25 patients for both outpatient settings (Figure 2) and inpatient settings (Figure 3).

### Guideline familiarity and adherence

**Figure 4.**
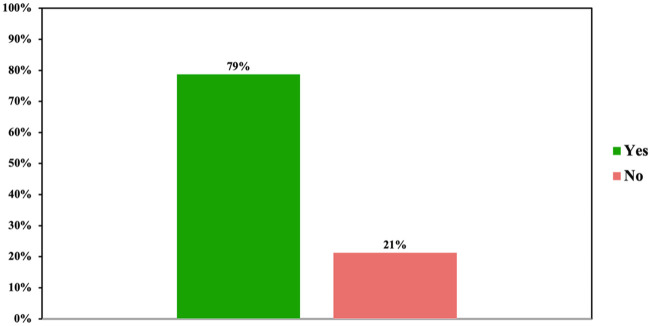
Guideline familiarity. Among all participants 79% demonstrated familiarity with German guideline recommendations regarding clozapine initiation.

Objective guideline familiarity was assessed based on participant’s knowledge regarding national guideline recommendations for clozapine initiation. A total of 79% of all participants (81% of residents (*n* = 13) and 78% of board-certified psychiatrists (*n* = 109)) demonstrated familiarity with German guideline recommendations regarding TRS criteria and appropriate timing for initiating clozapine (Figure 4). These recommendations are identical to international consensus recommendations.^5,59,134^

#### Timing of clozapine initiation

**Figure 5.**
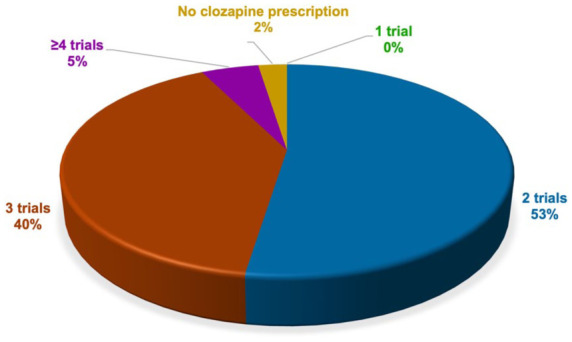
Preferred number of antipsychotic treatment trials before clozapine among prescribers familiar with national guidelines. Among prescribers familiar with current national guideline recommendations (*n* = 122), 53% reported initiating clozapine after two unsuccessful trials with standard antipsychotics, while 40% reported initiating clozapine after three unsuccessful trials with standard antipsychotics, 5% reported initiating clozapine after four or more unsuccessful trials with standard antipsychotics, and 2% reported not prescribing clozapine.

Among participants indicating familiarity with guideline recommendations (*n* = 122), 53% (*n* = 64) reported initiating clozapine after two other unsuccessful antipsychotic trials, 40% (*n* = 49) reported initiating clozapine after three other unsuccessful antipsychotic trials, 5% (*n* = 6) reported initiating clozapine after four or more other unsuccessful antipsychotic trials, and 2% (*n* = 3) reported not prescribing clozapine at all (Figure 5).

**Figure 6.**
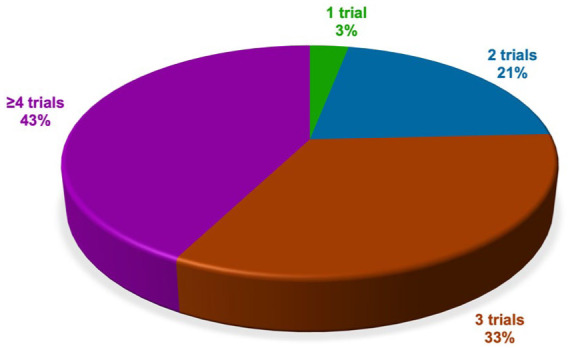
Preferred number of antipsychotic treatment trials before clozapine among prescribers unfamiliar with national guidelines. Among prescribers unfamiliar with current national guideline recommendations (*n* = 33), 21% reported initiating clozapine after two unsuccessful trials with standard antipsychotics, 33% reported initiating clozapine after three unsuccessful trials with standard antipsychotics, and 43% reported initiating clozapine after four unsuccessful trials with standard antipsychotics.

Among participants unfamiliar with guideline recommendations (*n* = 33), 3% (*n* = 1) reported initiating clozapine after one other unsuccessful trial, 21% (*n* = 7) reported initiating clozapine after two other unsuccessful antipsychotic trials, 33% (*n* = 11) reported initiating clozapine after three other unsuccessful antipsychotic trials, and 43% (*n* = 14) reported initiating clozapine after four or more other antipsychotic trials (Figure 6).

Among participants with supervised clozapine use only, 47.4% reported to initiate clozapine after two unsuccessful antipsychotic trials. In contrast, among participants with additional formalized training, 45.3% reported to initiate clozapine after two unsuccessful antipsychotic trials. The Pearson Chi-squared test did not reveal any significant effect of training type on the timing of clozapine initiation (χ^2^(1) = 0.059, *p* = 0.808, φ = 0.021).

#### Antipsychotic polypharmacy prior to clozapine

Notably, 72% of all participants reported conducting at least one trial of polypharmacy with standard antipsychotics prior to clozapine contrary to German guideline recommendations. Among the 122 participants familiar with German guideline recommendations, 69% reported conducting at least one trial of polypharmacy with standard antipsychotics prior to clozapine contrary to guideline recommendations. Among the 33 participants unfamiliar with German guideline recommendations, this rate was 82%.

Participants who had completed formalized training in clozapine management in addition to supervised use appeared to be less likely to favor the use of antipsychotic polypharmacy before initiating clozapine. Among participants with supervised clozapine use only, 76% reported a preference for polypharmacy prior to clozapine. In contrast, among participants with additional formalized training, 66% reported a preference for polypharmacy prior to clozapine. However, the Pearson Chi-squared test did not reveal any significant effect of additional formalized training on polypharmacy preference (χ^2^(1) = 1.946, *p* = 0.163, φ = 0.118).

### Confidence in clozapine use

A total of 97% of all participants reported feeling confident in monitoring patients on clozapine and 94% of all participants reported feeling confident in managing clozapine-associated ADRs. Among participants who expressed confidence in monitoring patients on clozapine (*n* = 151), the majority (*n* = 141) had acquired experience in clozapine management either through supervised use (*n* = 74) or supervised use and additional formalized training (*n* = 63). Among participants who expressed confidence in managing clozapine-associated ADRs (*n* = 146), the majority (*n* = 136) had acquired experience in clozapine management either through supervised use (*n* = 70) or supervised use and additional formalized training (*n* = 62).

### Knowledge regarding clozapine’s effectiveness

**Figure 7.**
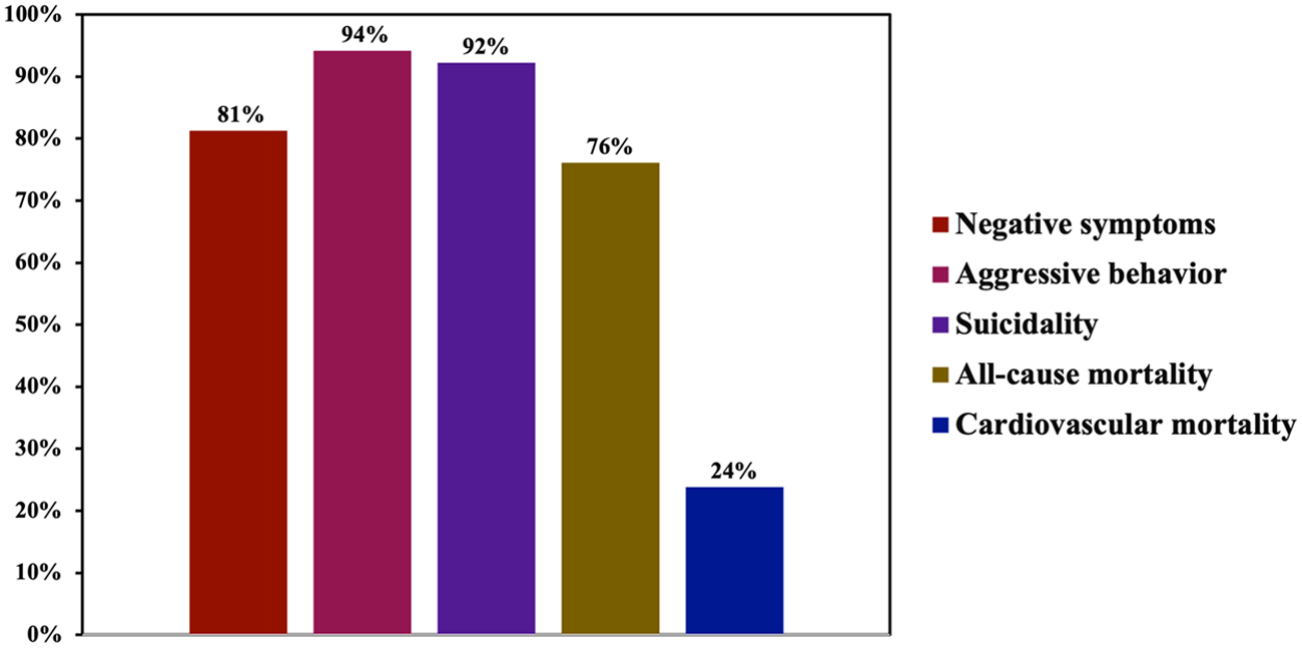
Reports about knowledge regarding clozapine effectiveness on key clinical parameters. Among all participants, the majority acknowledged that clozapine is effective in reducing negative symptoms (81%), aggressive behavior (94%), and suicidality (92%). Notably, 76% acknowledged that clozapine reduces all-cause mortality, whereas only 24% acknowledged that clozapine reduces cardiovascular mortality despite its potential metabolic ADRs. ADR, adverse drug reaction.

For descriptive purposes, we dichotomized the responses into “agreement” (“Yes” and “Likely Yes”) and “disagreement” (“No” and “Likely No”). Overall, among all participants, the majority acknowledged that clozapine is effective in reducing negative symptoms (81%), aggressive behavior (94%), and suicidality (92%) (Figure 7). Notably, 76% of participants acknowledged that clozapine reduces all-cause mortality. In contrast, only 24% of participants acknowledged that clozapine could reduce cardiovascular mortality despite its potential ADRs. Among participants who stated correctly that clozapine can reduce cardiovascular mortality (*n* = 37), 89% had acquired experience in clozapine management either through supervised use or supervised use and additional formalized training.

### Impact of additional formalized training on knowledge

To assess the impact of training-type using the original four-point-Likert-type responses, we conducted Mann–Whitney *U* tests (Table 3).

**Table 3.** Impact of additional formal training on knowledge regarding clozapine’s effectiveness.

Outcome	Supervised use only			Supervised use and additional formalized training			Test statistics		
	Median	Interquartile range (IQR)	Score reflecting	Median	Interquartile range (IQR)	Score reflecting	*U*	*Z*	*p*-Value
Negative symptoms	2	2	~“Likely Yes”	1	1	~“Yes”	1670.0	−3.47	<0.001
Aggressive behavior	2	1	~“Likely Yes”	1	1	~“Yes”	1887.5	2.58	0.010
Suicidality	2	1	~“Likely Yes”	1	1	~“Yes”	1780.0	−3.11	0.002
All-cause mortality	2	2	~“Likely Yes”	2	2	~“Likely Yes”	2208.5	−1.00	0.315
Cardiovascular mortality	3	1	~“Likely No”	3	0	~“Likely No”	1859.5	−2.57	0.01

#### Negative symptoms

For negative symptoms, the median rating was 2 (IQR = 2; ~“Likely Yes”) among participants with supervised use only, compared to 1 (IQR = 1; ~“Yes”) among participants with additional formalized training. The Mann–Whitney *U* test revealed a statistically significant effect of additional formalized training, *U* = 1670.0, *Z* = −3.47, *p* < 0.001.

#### Aggressive behavior

For aggressive behavior, the median rating was 2 (IQR = 1; ~“Likely Yes”) among participants with supervised use only, compared to 1 (IQR = 1; ~“Yes”) among participants with additional formalized training. The Mann–Whitney *U* test revealed a statistically significant effect of additional formalized training, *U* = 1887.5, *Z* = 2.58, *p* = 0.010.

#### Suicidality

For suicidality, the median rating was 2 (IQR = 1; ~“Likely Yes”) among participants with supervised use only, compared to 1 (IQR = 1; ~“Yes”) among participants with additional formalized training. The Mann–Whitney *U* test revealed a statistically significant effect of additional formalized training, *U* = 1780.0, *Z* = −3.11, *p* = 0.002.

#### All-cause mortality

For all-cause mortality, the median rating was 2 (IQR = 2; ~“Likely Yes”) in both the supervised-use-only group and the group with additional formalized training. The Mann–Whitney *U* test did not reveal a statistically significant effect of additional formalized training, *U* = 2208.5, *Z* = −1.00, *p* = 0.315.

#### Cardiovascular mortality

For cardiovascular mortality, the median rating was 3 (IQR = 1; between ~“Likely No”) in both the supervised-use-only group and the group with additional formalized training. However, the responses differed in their overall distribution, with the Mann–Whitney *U* test revealing a statistically significant effect of additional formalized training, *U* = 1859.5, *Z* = −2.57, *p* = 0.01.

### Assumed patient preferences

**Figure 8.**
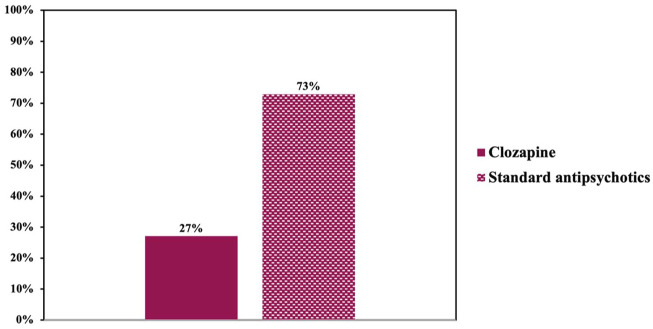
Presumed patients’ preferences. Two-thirds of the participants reported assuming that patients would prefer standard antipsychotics over clozapine.

For descriptive purposes, we first dichotomized the responses to the question whether patients would prefer clozapine over standard antipsychotics into “agreement” (“Yes” and “Likely Yes”) and “disagreement” (“No” and “Likely No”). A total of 73% of all participants assumed that patients would prefer standard antipsychotics over clozapine (Figure 8). Among participants without additional formalized training in clozapine use, this rate was 80%. Among participants with additional formalized training in clozapine use, this rate was 63%.

To assess the impact of training type using the original four-point-Likert-type responses, we conducted Mann–Whitney *U* tests. Overall, the median rating was 3 (IQR = 0; ~“Likely No”) among participants with supervised use only, compared to 3 (IQR = 1; ~“Likely No”) among participants with additional formalized training. However, the responses differed in their overall distribution, with the *Mann-Whitney-U* test revealing a statistically significant effect of additional formalized training, *U* = 1927.5, *Z* = −2.36, *p* = 0.018.

### General barriers for clozapine use

**Figure 9.**
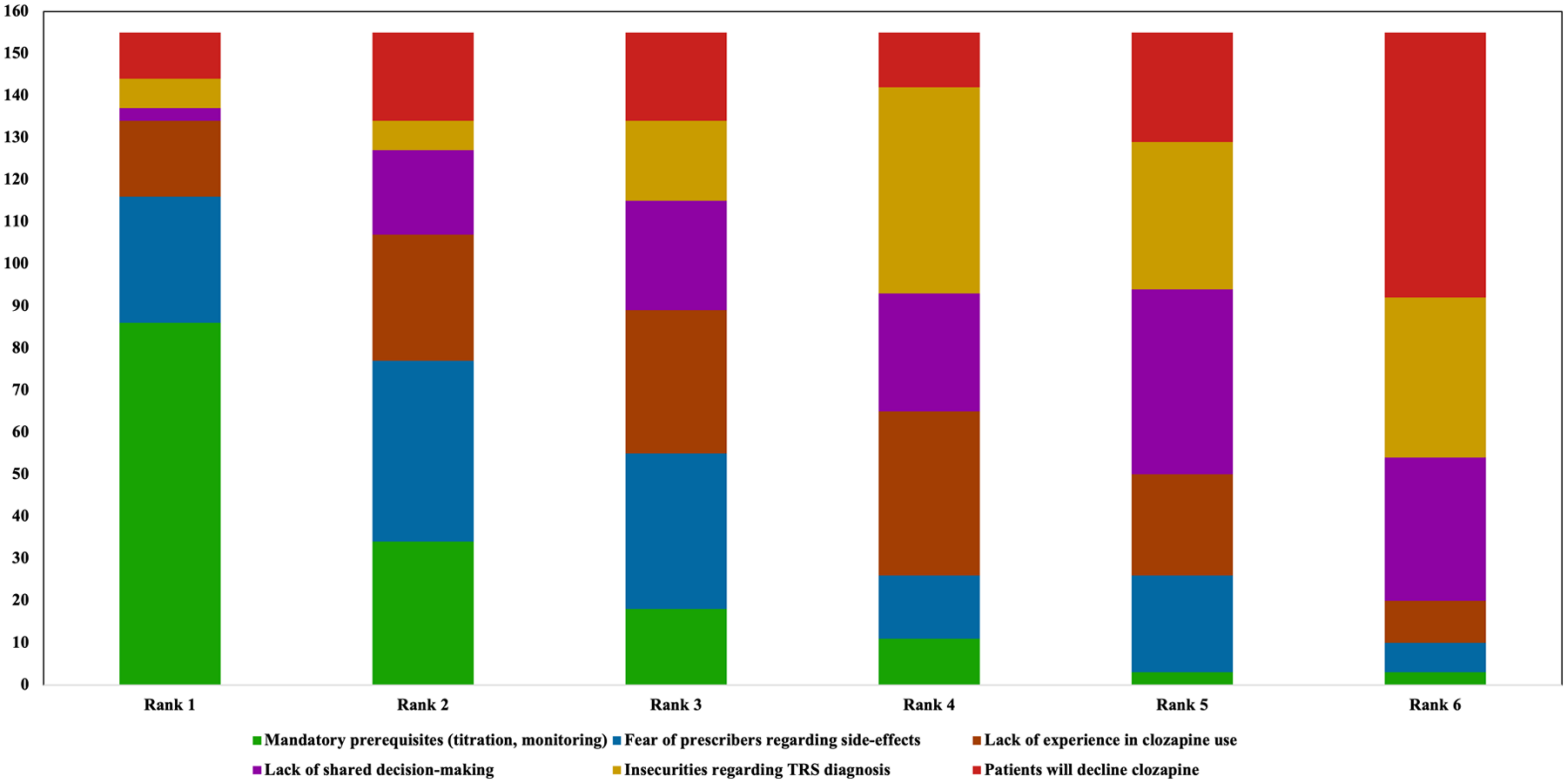
Ranking of general barriers limiting clozapine use. Participants ranked the mandatory treatment prerequisites of clozapine, including monitoring requirements and slow titration, as the most relevant treatment barriers. Concerns regarding ADRs and lack of experience in clozapine use were reported as additional relevant barriers. Uncertainties regarding TRS diagnosis, lack of shared decision-making, and potential patient refusal of clozapine were less frequently reported as barriers. ADR, adverse drug reaction; TRS, treatment-resistant schizophrenia.

All participants were asked to rank factors they perceived as barriers in order of relevance (Figure 9), with one reflecting the most important barrier. Here, mandatory prerequisites, including monitoring requirements and slow titration, were ranked highest overall. Specifically, 86 participants assigned mandatory prerequisites to the first rank. Concerns about ADRs (~ second rank) and lack of experience of psychiatric prescribers in general (~ third rank) in clozapine use were regarded as additional important factors limiting clozapine use. Insecurities regarding TRS diagnosis (~ fourth rank), lack of shared decision-making (~ fifth rank), and potential patient refusal (~ sixth rank) of clozapine were less frequently considered as relevant barriers.

### ADR-related barriers for clozapine use

**Figure 10.**
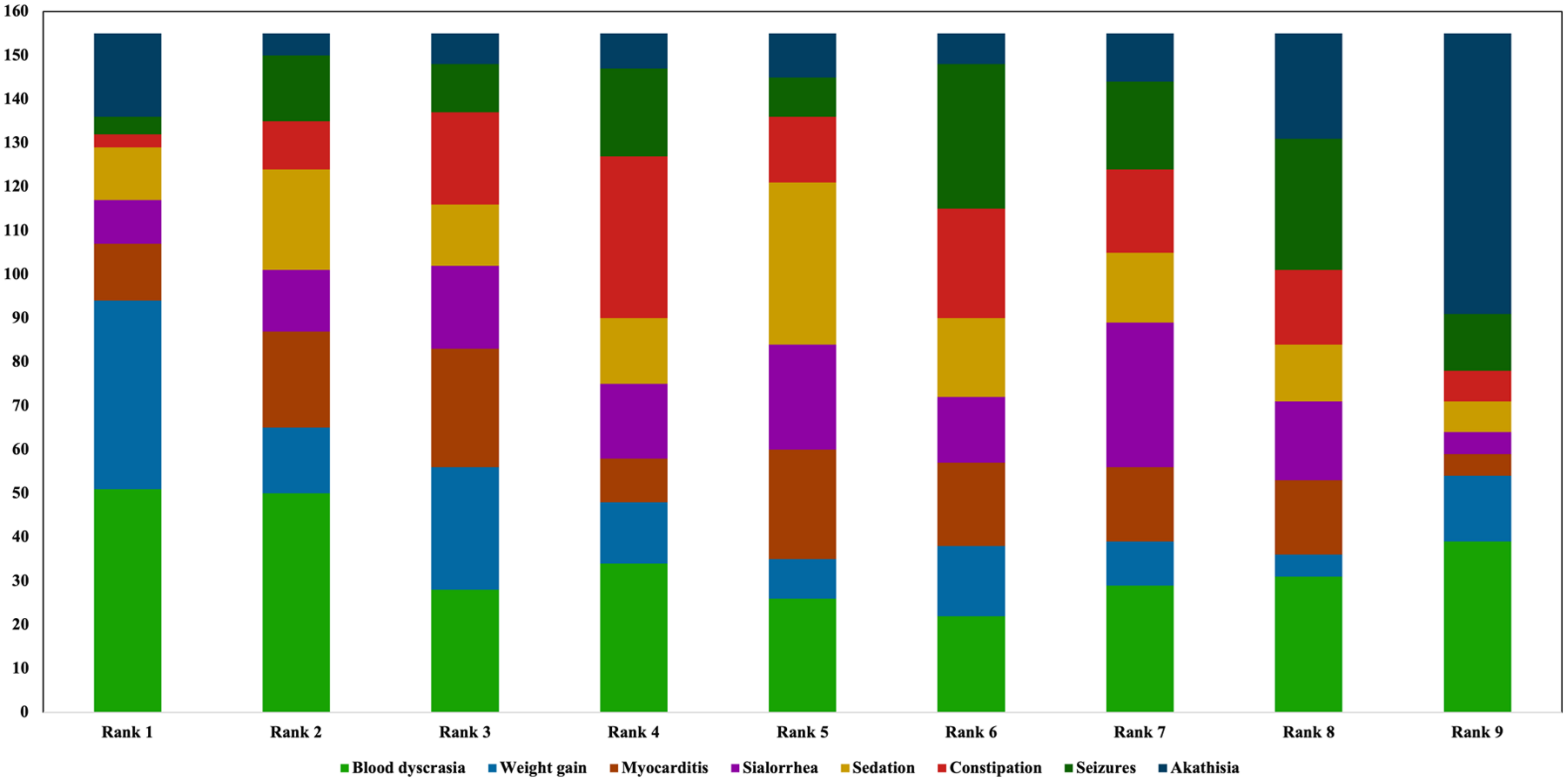
Ranking of ADR-related barriers limiting clozapine use. Participants ranked blood dyscrasia as the most relevant ADR limiting clozapine-use, closely followed by clozapine-associated weight gain, sedation, myocarditis, and sialorrhea. Constipation was less frequently reported as a barrier, followed by seizures and akathisia. ADR, adverse drug reaction.

All participants were asked to rank clozapine-associated ADRs they perceived as a barrier for clozapine long-term use in order of relevance (Figure 10), with one reflecting the most important barrier. Here, blood dyscrasia was ranked highest (~ first rank). This was closely followed by clozapine-associated weight gain, sedation, myocarditis, and sialorrhea. Constipation (~ fifth rank) was less frequently considered as a barrier. This was closely followed by seizures and akathisia.

### Patient-related barriers for clozapine use

**Figure 11.**
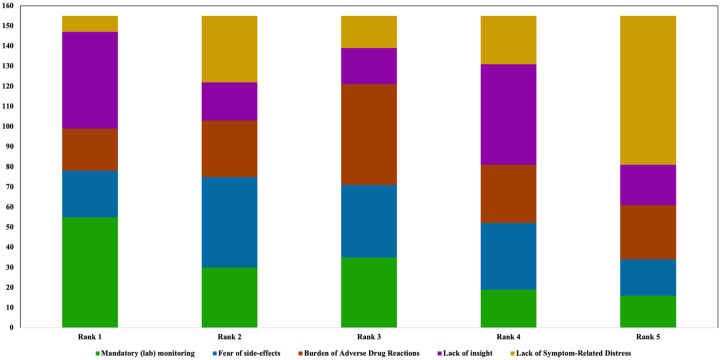
Ranking of barriers limiting clozapine use from the patient perspective. Participants ranked mandatory laboratory monitoring and concerns regarding ADRs as the most relevant factors from the patients’ perspective limiting clozapine use, followed by patients’ perceived ADR burden. Patients’ lack of insight and lack of symptom-related distress was less frequently reported as relevant barriers. ADR, adverse drug reaction.

Participants were asked to rank factors they assumed would limit clozapine use from the patients’ perspective in order of relevance (Figure 11), with one reflecting the most important factor. Here, mandatory laboratory monitoring was ranked highest (~ first rank). Concerns about ADRs were assigned to the second rank. The perceived burden of ADRs was predominantly assigned to the third rank. Lack of insight was ranked first by a considerable number of participants (*n* = 48), but overall, it was assigned to the fourth rank (*n* = 50). Interestingly, a lack of symptom-related distress was less frequently considered as a relevant barrier (~ sixth rank).

## Discussion

Our goal was to examine prescriber-related factors for the considerable underutilization of clozapine in schizophrenia in Germany despite its status as the only antipsychotic with proven efficacy in patients with TRS.^5,11–15^ To this end, we used an online survey to examine attitudes toward clozapine and factors limiting the adequate use of clozapine in a sample of 155 participants.

### Comparability with other studies

While our study constitutes the first assessment of the situation in Germany, a growing number of studies across the globe have evaluated psychiatrists’ attitudes toward clozapine.^61–85^ Our participants’ age range and professional profiles mirrored those reported in similar surveys.^66–70,74,77,85^

### Main findings

Our main findings were that monitoring requirements and concerns regarding ADRs are most frequently regarded as factors limiting more widespread clozapine use in Germany. Interestingly, while participants rated themselves as sufficiently experienced in the use of clozapine, they also regarded a lack of experience with clozapine among psychiatric prescribers in general as an almost equally important treatment barrier. Limited adherence to guideline recommendations regarding clozapine initiation and a lack of knowledge regarding the positive impact of clozapine on cardiovascular mortality despite its metabolic side-effects also emerged as potential barriers. Furthermore, participants clearly presumed that patients would prefer standard antipsychotics over clozapine.

### ADR-related concerns

Concerns regarding blood dyscrasia, that is, CIN and CIA, were ranked most frequently as ADR-related barriers followed by weight gain, while constipation and clozapine-induced myocarditis (CIM) were named less frequently. These assessments contrast with current evidence indicating that the risk of CIM might be notably higher than the risk of CIA.^95,137–139^

### Monitoring requirements

Importantly, we conducted our study before the European Medicines Agency and the German Federal Institute for Drugs and Medical Devices eased blood monitoring requirements in September 2025 following a concerted effort by the European Clozapine Task Force.^95,140,141^ Future studies should investigate the impact of these changes, which were partly motivated by the positive experience of reduced blood monitoring during the COVID-19 pandemic,^142,143^ on prescriber attitudes and prescription rates.

### Titration speed

The slow titration speed required during clozapine initiation also appeared to be regarded as a barrier.^144^ However, because this potential barrier was assessed together with laboratory monitoring, we cannot draw definitive conclusions about whether it was considered as a major barrier on its own. Considering the current state of evidence,^61–68,70–76,78–85,145^ we would assume that laboratory monitoring would have a higher impact on participants’ treatment decisions than slow titration. Given that the vast majority of studies has demonstrated an association of rapid initial titration rates—even in the order of 25 mg per day—with an increased risk for severe ADRs, particularly CIM, recent expert consensus recommendations clearly advise against such fast titration regimes.^138,144,146–150^ Moreover, the occurrence of other ADRs induced by rapid titration rates might compromise long-term treatment adherence.^25,138^ Therefore, psychiatric training in the context of TRS treatment needs to emphasize pharmacological strategies suited for stabilizing acutely ill TRS patients until the clinical effects of clozapine can become evident, such as the use of olanzapine and benzodiazepines.

### Clozapine initiation

#### Timing of clozapine initiation

Among the 79% of participants familiar with German guideline recommendations regarding the timing of clozapine initiation, 45% reported deviating from this recommendation by typically initiating clozapine after at least three other unsuccessful antipsychotic trials. Among the 21% of participants unfamiliar with these recommendations, 76% reported typically initiating clozapine after at least three other unsuccessful antipsychotic trials. Additional formalized training in clozapine use did not increase the likelihood of initiating clozapine after two unsuccessful antipsychotic trials.

#### Antipsychotic polypharmacy prior to clozapine

Notably, contrary to German guideline recommendations, 72% of all participants reported conducting at least one trial of polypharmacy with standard antipsychotics within the sequence of pre-clozapine antipsychotic trials. For this issue, we did not observe a significant effect of additional formalized training in clozapine use.

Among participants familiar with German guideline recommendations, this rate was 69%, while it was 82% among participants unfamiliar with German guideline recommendations. Thus, while guideline familiarity appears to have a discernable positive impact on clozapine initiation behavior, the overall rate of deviation from the recommendations remained considerable. These findings are well in line with prescribing patterns reported by other studies.^67–69,74,82,151–154^

Importantly, our survey did not allow us to determine at which point during pre-clozapine antipsychotic trials antipsychotic polypharmacy was typically used. However, our findings indicate that even among prescribers who initiate clozapine after two other antipsychotic trials, the majority employ an antipsychotic polypharmacy strategy prior to clozapine. Importantly, we explicitly asked whether this involves the use of both antipsychotics in a dose deemed to have antipsychotic efficacy. We interpret this finding as indicating that antipsychotic polypharmacy is relatively common as a second-line treatment strategy. This finding would constitute one of the most concerning and puzzling deviations from evidence-based antipsychotic prescribing for schizophrenia. However, given that we assessed polypharmacy prior to clozapine initiation and the timing of clozapine initiation more generally in two independent questions, we cannot rule out some level of inconsistency in the way both questions were answered.

### Clozapine’s effectiveness

The impact of clozapine on key outcome parameters beyond positive symptoms represents an important aspect of its overall effectiveness.^25^ In our study, a clear majority of participants acknowledged clozapine’s effectiveness in reducing negative symptoms, aggressive behavior, suicidality, and all-cause mortality. In contrast, and contrary to the existing evidence,^19,33–36^ only 24% of participants recognized its association with reduced cardiovascular mortality—a finding of particular relevance given widespread concerns about clozapine’s metabolic and cardiac ADRs. With the exception of all-cause mortality, additional formalized training in clozapine use was associated with significantly greater awareness of these outcome benefits.

### Assumed patient preferences

Importantly, the majority of participants assumed that patients would prefer standard antipsychotics over clozapine, a finding consistent with previous surveys.^68,69,71,74^ However, converging evidence shows that despite potential ADRs—particularly hypersalivation and weight gain—and the burden of mandatory monitoring, patients treated with clozapine report higher satisfaction than with their prior standard antipsychotic treatment.^71,81,86,87,90^

Together, these findings suggest that clozapine underutilization is primarily driven by prescriber concerns regarding ADRs and monitoring requirements. Previous work indicates that such concerns may be exacerbated by limited familiarity with treatment algorithms and ADR management strategies,^64,65,72,155^ potentially creating a self-reinforcing cycle in which low prescribing rates lead to less clinical expertise.^64,65,72,89,155^ These factors may further increase the likelihood that clozapine is not even offered as a treatment option, thereby excluding patients from shared decision-making regarding a uniquely effective treatment option with unrivaled benefits.^89^

### Possible solutions for clozapine underutilization

#### Shared decision-making

We did not specifically assess the frequency of use of shared decision-making (SDM) in our survey. However, there are clear recommendations for its consistent use in treating patients with schizophrenia,^156–159^ because it increases the impact of the patients’ perspective and needs.^159^ Moreover, emphasizing the patient’s perspective and their need for adequate treatment increases the likelihood of psychiatric residents considering and recommending clozapine.^89,156,160^ Consequently, focusing on these issues could be a crucial catalyst of clozapine use. Importantly, implementing SDM should also involve people with lived experience (PWLE) who have benefited from clozapine.^145,161^

#### Training in clozapine use

Reframing how clozapine is presented in the undergraduate and post-graduate medical literature appears to be important. Many publications and textbooks still refer to clozapine as a “last-resort medication,” implying that clozapine should be used after non-response to all other antipsychotics despite clear guideline recommendations to the contrary. Emphasizing the unique advantages of clozapine during medical training, rather than focusing primarily on its ADRs might help to promote its wider use.^40^ Such a strategy should also include increased advocacy by networks involving both clinical experts and PWLE.

Given that a lack of experience in clozapine use was regarded as a relevant barrier by our participants, mandatory formalized training in clozapine use appears to be essential. In our cohort, it increased knowledge regarding the extent of clozapine’s effectiveness, while not noticeably improving guideline adherence. This suggests that existing training formats may insufficiently emphasize treatment algorithms, and particularly the prognostic relevance of minimizing delays in clozapine initiation. Strengthening this focus may enhance the impact of mandatory clozapine training as a requirement for board certification.^65^

Such mandatory training is widely regarded as a key component of comprehensive strategies to address clozapine underutilization.^40,162,163^ To ensure broad accessibility, e-learning and blended learning approaches might be most suitable.^163–166^ Beyond treatment algorithms, such programs should prioritize early detection of TRS, increasing awareness regarding patients’ positive attitudes toward clozapine, teaching SDM and effective ADR management, and highlighting the full range of clozapine’s effectiveness on clinical syndromes and mortality. However, it is essential to integrate such training programs with practical clinical training.^163^

#### TRS units

Establishing hospital-based TRS units across Germany comparable to the Maudsley TREAT team represents another strategy to enhance expertise in clozapine use and TRS detection.^167^ Ideally, these units should combine structured case referral pathways^168^ with case-specific consultation hotlines to support mental health professionals who lack direct access to specialized expertise, analogous to the “Medical Child Protection Hotline” coordinated by Ulm University Hospital.^169^ Given that approximately 80% of TRS cases emerge during the first psychotic episode,^6,8–10^ systematic integration of dedicated expertise in clozapine use and TRS detection into EIS appears essential.^170^ Ideally, EIS should be closely linked with specialized TRS units. Current evidence indicates that such an integrative approach can substantially reduce delays in clozapine initiation.^170,171^

#### Beyond clozapine

Finally, clozapine underutilization parallels the considerable underuse of lithium in bipolar disorder,^172–175^ despite lithium representing an equally indispensable treatment option for this condition.^173,174,176–178^ This parallel points to a more fundamental challenge in psychiatric practice: the insufficient implementation of highly effective, evidence-based pharmacological treatments with demanding monitoring requirements and complex ADR management. Addressing this challenge may, therefore, require structural changes in postgraduate training and continuing medical education that extend beyond disorder-specific approaches.

### Limitations

Some limitations of our survey need to be considered. First, we cannot determine whether the participants were aware of the concurrent efforts by European clozapine researchers advocating a relaxation of blood-monitoring^83^ and whether that may have influenced their attitudes. More importantly, despite our efforts to reach most prescribers by contacting all major professional networks, only 155 psychiatrists completed the survey. We cannot make any inferences as to why many individuals contacted did not complete the survey. Thus, we cannot tell whether the degree of professional personal experience in using clozapine influenced the likelihood of participating in the study. Consequently, an important limitation of this study is the absence of an a-priori sample size calculation or formal power analysis. This survey was designed as an exploratory, cross-sectional study and was distributed anonymously via national professional psychiatric networks without access to a defined sampling frame. As a result, neither the total number of eligible psychiatrists approached nor a single primary outcome with an anticipated effect size could be specified in advance. This precluded a meaningful a-priori power-based sample size estimation and reliable calculation of response rates.

Moreover, only 23% of the participants worked in private practices. Consequently, there might be a selection bias regarding self-reported clozapine prescription patterns and confidence in clozapine management. However, given that to the best of our knowledge no study has previously investigated prescriber-related barriers to clozapine use in Germany, our findings should still be of use to improve strategies for increasing clozapine prescription rates.

Additionally, 90% of the participants were already board-certified and almost all participants reported being regular clozapine prescribers. This might have also contributed to the high rates of self-reported clozapine confidence and the relatively high familiarity with national guideline recommendations. While this could indicate a bias in our participants toward experienced and regular prescribers, this could also be regarded as a strength. This cohort should provide particularly informed insights into clozapine use. However, considering the evident need to improve and formalize clozapine training during psychiatric residency, additional studies specifically targeting early-career residents are warranted to complement and extend our current findings.

## Conclusion

In line with previous surveys from other countries, our findings show that despite familiarity with national guideline recommendations, psychiatrists are reluctant to prescribe clozapine. Barriers leading to delayed clozapine use encompass concerns about tolerability, logistical issues related to mandatory blood monitoring, and misconceptions regarding patients’ preferences. However, additional formalized training including shared decision-making tools combined with supervised clozapine prescription during residency could increasing clozapine prescription rates.

Future research should investigate the benefits of such training programs, the effects of the recently eased blood monitoring requirements, as well as the impact of integrating PWLE advocating regular use of clozapine. Moreover, research on clozapine perspectives needs to identify and address remaining gaps in knowledge.^71^ These include attitudes of clozapine-eligible but naïve patients, clinicians’ reasons for withholding clozapine and factors facilitating its use, as well as prescriber attitudes toward clozapine continuation, discontinuation, and rechallenge.^71^ Such studies will be critical to overcoming one of the most enduring and clinically consequential gaps in the implementation of evidence-based psychiatric care.

## Supplemental Material

sj-docx-1-tpp-10.1177_20451253261434380 – Supplemental material for Adult psychiatrists’ views on clozapine prescribing for schizophrenia in Germany—an online survey Supplemental material, sj-docx-1-tpp-10.1177_20451253261434380 for Adult psychiatrists’ views on clozapine prescribing for schizophrenia in Germany—an online survey by Mishal Qubad, Ida Marie Ehret, Christian J. Bachmann and Robert A. Bittner in Therapeutic Advances in Psychopharmacology

sj-docx-2-tpp-10.1177_20451253261434380 – Supplemental material for Adult psychiatrists’ views on clozapine prescribing for schizophrenia in Germany—an online survey Supplemental material, sj-docx-2-tpp-10.1177_20451253261434380 for Adult psychiatrists’ views on clozapine prescribing for schizophrenia in Germany—an online survey by Mishal Qubad, Ida Marie Ehret, Christian J. Bachmann and Robert A. Bittner in Therapeutic Advances in Psychopharmacology

## Declarations

**Ethics approval and consent to participate:** The study protocol was approved by the ethical review board of the Faculty of Medicine at Goethe University, which granted a waiver for this study (reference number: 2024-1787). All participants indicated their informed consent by selecting a consent checkbox at the beginning which was required for starting the survey.

**Consent for publication:** Not applicable.

**Availability of data and materials:** The data that support the findings of this study are available from the corresponding author upon reasonable request.
