# Supplementary material for: Adult psychiatrists’ views on clozapine prescribing for schizophrenia in Germany—an online survey
Source: Ther Adv Psychopharmacol. 2026 Apr 17;16:20451253261434380. doi: 10.1177/20451253261434380 (PMC13100437; doi:10.1177/20451253261434380)
Supplement: sj-docx-2-tpp-10.1177_20451253261434380 – Supplemental material for Adult psychiatrists’ views on clozapine prescribing for schizophrenia in Germany—an online survey [file sj-docx-2-tpp-10.1177_20451253261434380.docx]

1. Bitte geben Sie Ihr Geschlecht an.

- Männlich

- Weiblich

- Divers

2. Bitte geben Sie aktuelles Alter an.

- < 20 Jahre

- 20-25 Jahre

- 26-30 Jahre

- 31-35 Jahre

- 36-40 Jahre

- 41-45 Jahre

- 46-50 Jahre

- 51-55 Jahre

- 56-60 Jahre

- 61-65 Jahre

- > 65 Jahre

3. Bitte geben Sie die Dauer Ihrer beruflichen Erfahrung im Bereich Psychiatrie und Psychotherapie an (inkl. ärztliche Weiterbildungszeit).

- <1 Jahr

- 1-2 Jahre

- 3-4 Jahre

- 5-10 Jahre

- 11-15 Jahre

- 16-20 Jahre

- 21-25 Jahre

- 26-30 Jahre

- 31-35 Jahre

- 36-40 Jahre

- 41-45 Jahre

- > 45 Jahre

4. Bitte geben Sie an, in welcher Einrichtung Sie Ihre Weiterbildung zur Fachärztin/zum Facharzt für Psychiatrie und Psychotherapie abgeschlossen haben.

- Ich bin noch StudentIn

- Ich befinde mich aktuell noch in der Weiterbildung

- Universitätsklinik mit Versorgungspflicht

- Universitätsklinik ohne Versorgungspflicht

- Nicht-universitäres Krankenhaus mit Versorgungspflicht

- Nicht-universitäres Krankenhaus ohne Versorgungspflicht

5. Bitte geben Sie an, in welchem Land Sie den überwiegenden Teil Ihres Studiums abgeschlossen haben.

- Afghanistan

- Ägypten

- Albanien

- Algerien

- Andorra

- Angola

- Antigua und Barbuda

- Äquatorialguinea

- Argentinien

- Armenien

- Aserbaidschan

- Äthiopien

- Australien

- Bahamas

- Bahrain

- Bangladesch

- Barbados

- Belgien

- Belize

- Benin

- Bhutan

- Bolivien

- Bosnien und Herzegowina

- Botsuana

- Brasilien

- Brunei

- Bulgarien

- Burkina Faso

- Burundi

- Chile

- China

- Costa Rica

- Dänemark

- Deutschland

- Dominica

- Dominikanische Republik

- Dschibuti

- Ecuador

- El Salvador

- Elfenbeinküste

- Eritrea

- Estland

- Eswatini

- Fidschi

- Finnland

- Frankreich

- Gabun

- Gambia

- Georgien

- Ghana

- Grenada

- Griechenland

- Guatemala

- Guinea

- Guinea-Bissau

- Guyana

- Haiti

- Honduras

- Indien

- Indonesien

- Irak

- Iran

- Irland

- Island

- Israel

- Italien

- Jamaika

- Japan

- Jemen

- Jordanien

- Kambodscha

- Kamerun

- Kanada

- Kap Verde

- Kasachstan

- Katar

- Kenia

- Kirgisistan

- Kiribati

- Kolumbien

- Komoren

- Kongo, Demokratische Republik

- Kongo, Republik

- Korea, Nord

- Korea, Süd

- Kosovo

- Kroatien

- Kuba

- Kuwait

- Laos

- Lesotho

- Lettland

- Libanon

- Liberia

- Libyen

- Liechtenstein

- Litauen

- Luxemburg

- Madagaskar

- Malawi

- Malaysia

- Malediven

- Mali

- Malta

- Marokko

- Marshallinseln

- Mauretanien

- Mauritius

- Mexiko

- Mikronesien

- Moldau

- Monaco

- Mongolei

- Montenegro

- Mosambik

- Myanmar

- Namibia

- Nauru

- Nepal

- Neuseeland

- Nicaragua

- Niederlande

- Niger

- Nigeria

- Nordmazedonien

- Norwegen

- Oman

- Österreich

- Osttimor

- Pakistan

- Palau

- Panama

- Papua-Neuguinea

- Paraguay

- Peru

- Philippinen

- Polen

- Portugal

- Ruanda

- Rumänien

- Russland

- Salomonen

- Sambia

- Samoa

- San Marino

- São Tomé und Príncipe

- Saudi-Arabien

- Schweden

- Schweiz

- Senegal

- Serbien

- Seychellen

- Sierra Leone

- Simbabwe

- Singapur

- Slowakei

- Slowenien

- Somalia

- Spanien

- Sri Lanka

- St. Kitts und Nevis

- St. Lucia

- St. Vincent und die Grenadinen

- Südafrika

- Sudan

- Südsudan

- Suriname

- Syrien

- Tadschikistan

- Tansania

- Thailand

- Togo

- Tonga

- Trinidad und Tobago

- Tschad

- Tschechien

- Tunesien

- Türkei

- Turkmenistan

- Tuvalu

- Uganda

- Ukraine

- Ungarn

- Uruguay

- Usbekistan

- Vanuatu

- Vatikanstadt

- Venezuela

- Vereinigte Arabische Emirate

- Vereinigte Staaten

- Vereinigtes Königreich

- Vietnam

- Weißrussland

- Zentralafrikanische Republik

- Zypern

6. Bitte geben Sie an, in welchem Land Sie den überwiegenden Teil Ihrer Weiterbildung zur Fachärztin/ zum Facharzt für Psychiatrie und Psychotherapie abgeschlossen haben.

- Afghanistan

- Ägypten

- Albanien

- Algerien

- Andorra

- Angola

- Antigua und Barbuda

- Äquatorialguinea

- Argentinien

- Armenien

- Aserbaidschan

- Äthiopien

- Australien

- Bahamas

- Bahrain

- Bangladesch

- Barbados

- Belgien

- Belize

- Benin

- Bhutan

- Bolivien

- Bosnien und Herzegowina

- Botsuana

- Brasilien

- Brunei

- Bulgarien

- Burkina Faso

- Burundi

- Chile

- China

- Costa Rica

- Dänemark

- Deutschland

- Dominica

- Dominikanische Republik

- Dschibuti

- Ecuador

- El Salvador

- Elfenbeinküste

- Eritrea

- Estland

- Eswatini

- Fidschi

- Finnland

- Frankreich

- Gabun

- Gambia

- Georgien

- Ghana

- Grenada

- Griechenland

- Guatemala

- Guinea

- Guinea-Bissau

- Guyana

- Haiti

- Honduras

- Indien

- Indonesien

- Irak

- Iran

- Irland

- Island

- Israel

- Italien

- Jamaika

- Japan

- Jemen

- Jordanien

- Kambodscha

- Kamerun

- Kanada

- Kap Verde

- Kasachstan

- Katar

- Kenia

- Kirgisistan

- Kiribati

- Kolumbien

- Komoren

- Kongo, Demokratische Republik

- Kongo, Republik

- Korea, Nord

- Korea, Süd

- Kosovo

- Kroatien

- Kuba

- Kuwait

- Laos

- Lesotho

- Lettland

- Libanon

- Liberia

- Libyen

- Liechtenstein

- Litauen

- Luxemburg

- Madagaskar

- Malawi

- Malaysia

- Malediven

- Mali

- Malta

- Marokko

- Marshallinseln

- Mauretanien

- Mauritius

- Mexiko

- Mikronesien

- Moldau

- Monaco

- Mongolei

- Montenegro

- Mosambik

- Myanmar

- Namibia

- Nauru

- Nepal

- Neuseeland

- Nicaragua

- Niederlande

- Niger

- Nigeria

- Nordmazedonien

- Norwegen

- Oman

- Österreich

- Osttimor

- Pakistan

- Palau

- Panama

- Papua-Neuguinea

- Paraguay

- Peru

- Philippinen

- Polen

- Portugal

- Ruanda

- Rumänien

- Russland

- Salomonen

- Sambia

- Samoa

- San Marino

- São Tomé und Príncipe

- Saudi-Arabien

- Schweden

- Schweiz

- Senegal

- Serbien

- Seychellen

- Sierra Leone

- Simbabwe

- Singapur

- Slowakei

- Slowenien

- Somalia

- Spanien

- Sri Lanka

- St. Kitts und Nevis

- St. Lucia

- St. Vincent und die Grenadinen

- Südafrika

- Sudan

- Südsudan

- Suriname

- Syrien

- Tadschikistan

- Tansania

- Thailand

- Togo

- Tonga

- Trinidad und Tobago

- Tschad

- Tschechien

- Tunesien

- Türkei

- Turkmenistan

- Tuvalu

- Uganda

- Ukraine

- Ungarn

- Uruguay

- Usbekistan

- Vanuatu

- Vatikanstadt

- Venezuela

- Vereinigte Arabische Emirate

- Vereinigte Staaten

- Vereinigtes Königreich

- Vietnam

- Weißrussland

- Zentralafrikanische Republik

- Zypern

7. Bitte geben Sie Ihren aktuellen Berufsstatus an. (Mehrfachauswahl möglich)

- Ich bin noch StudentIn

- Ärztin/Arzt in Weiterbildung für Psychiatrie und Psychotherapie

- Fachärztin/Facharzt für Psychiatrie und Psychotherapie/Nervenheilkunde

- Oberärztin/Oberarzt für Psychiatrie und Psychotherapie/Nervenheilkunde

- Chefärztin/Chefarzt bzw. KlinikdirektorIn einer Klinik für Psychiatrie und Psychotherapie/Nervenheilkunde

- Forschung

- Sonstige

8. Bitte geben Sie an, in welcher Einrichtung Sie aktuell arbeiten.

- Ich bin noch StudentIn

- Universitätsklinik mit Versorgungspflicht

- Universitätsklinik ohne Versorgungspflicht

- Nicht-universitäre psychiatrische Klinik mit Versorgungspflicht

- Nicht-universitäre psychiatrische Klinik ohne Versorgungspflicht

- Nicht-universitäre psychiatrische Abteilung an einem Allgemeinkrankenhaus mit Versorgungspflicht

- Nicht-universitäre psychiatrische Abteilung an einem Allgemeinkrankenhaus ohne Versorgungspflicht

- Niederlassung in einer psychiatrischen Praxis

- Primär psychotherapeutisch-orientierte Praxis

- Rehaklinik

- Neurologie (Niederlassung)

- Neurologie (Klinik)

- Sonstige

9. Bitte geben Sie an, in welchen Einrichtungen Sie während Ihrer Weiterbildung zur Fachärztin/zum Facharzt für Psychiatrie und Psychotherapie bereits gearbeitet haben. (Mehrfachauswahl möglich)

- Ich bin noch StudentIn oder in der Weiterbildung

- Universitätsklinik mit Versorgungspflicht

- Universitätsklinik ohne Versorgungspflicht

- Nicht-universitäre psychiatrische Klinik mit Versorgungspflicht

- Nicht-universitäre psychiatrische Klinik ohne Versorgungspflicht

- Nicht-universitäre psychiatrische Abteilung an einem Allgemeinkrankenhaus mit Versorgungspflicht

- Nicht-universitäre psychiatrische Abteilung an einem Allgemeinkrankenhaus ohne Versorgungspflicht

- Niederlassung in einer psychiatrischen Praxis

- Primär psychotherapeutisch-orientierte Praxis

- Rehaklinik

- Neurologie (Niederlassung)

- Neurologie (Klinik)

- Sonstige

10. Behandeln Sie oder haben Sie jemals PatientInnen mit Schizophrenie behandelt?

- Ja

- Nein

11. Wie viele PatientInnen mit einer Schizophrenie haben Sie ungefähr im Rahmen Ihrer gesamten Tätigkeit im Bereich der Psychiatrie behandelt?

- Keine

- 1-10 PatientInnen

- 11-25 PatientInnen

- 26-50 PatientInnen

- 51-100 PatientInnen

- 101-150 PatientInnen

- 151-200 PatientInnen

- 201-500 PatientInnen

- > 500 PatientInnen

12. Wie viele PatientInnen mit einer Schizophrenie haben Sie ungefähr im letzten Monat behandelt?

- Keine

- < 5 Patienten

- 6-10 Patienten

- 11-15 Patienten

- 16-20 Patienten

- 21-25 Patienten

- > 25 Patienten

13. Behandeln Sie aktuell PatientInnen mit Clozapin?

- Ja

- Nein

14. Bitte schätzen Sie die Anzahl Ihrer ambulanten PatientInnen, die derzeit mit Clozapin behandelt werden.

- Keine

- ich bin nicht ambulant tätig

- 1-10 PatientInnen

- 11-25 PatientInnen

- 26-50 PatientInnen

- 51-100 PatientInnen

- >101 PatientInnen

15. Bitte schätzen Sie die Anzahl Ihrer stationären PatientInnen, die derzeit mit Clozapin behandelt werden.

- Keine

- ich bin nicht stationär tätig

- 1-10 PatientInnen

- 11-25 PatientInnen

- 26-50 PatientInnen

- >50 PatientInnen

16. Haben Sie früher Clozapin verordnet?

- Ja

- Nein

17. Bitte schätzen Sie die Anzahl der bei Ihnen ambulant behandelten PatientInnen, bei denen aktuell eine Clozapinbehandlung indiziert wäre.

- Keine

- ich bin nicht ambulant tätig

- 1-5 PatientInnen

- 6-10 PatientInnen

- 11-15 PatientInnen

- 16-20 PatientInnen

- 21-25 PatientInnen

- 26-30 PatientInnen

- 31-35 PatientInnen

- 36-40 PatientInnen

- 41-45 PatientInnen

- 46-55 PatientInnen

- 56-65 PatientInnen

- 66-75 PatientInnen

- > 76 PatientInnen

18. Bitte schätzen Sie die Anzahl der bei Ihnen stationär behandelten PatientInnen, bei denen aktuell eine Clozapinbehandlung indiziert wäre.

- Keine

- ich bin nicht stationär tätig

- 1-5 PatientInnen

- 6-10 PatientInnen

- 11-15 PatientInnen

- 16-20 PatientInnen

- 21-25 PatientInnen

- 26-30 PatientInnen

- 31-35 PatientInnen

- 36-40 PatientInnen

- 41-45 PatientInnen

- 46-55 PatientInnen

- 56-65 PatientInnen

- 66-75 PatientInnen

- > 76 PatientInnen

19. Haben Sie jemals im Rahmen Ihrer Krankenhaustätigkeit (z.B. unter oberärztlicher Supervision) eine spezifische Anleitung zur Behandlung mit Clozapin erhalten?

- Ja

- Nein

20. Haben Sie den Eindruck, dass diese Anleitung ausreichend war?

- Ja

- Nein

21. Haben Sie jemals eine spezifische externe Anleitung (z.B. eine Fortbildung) zur Behandlung mit Clozapin erhalten?

- Ja

- Nein

22. Haben Sie den Eindruck, dass diese Anleitung ausreichend war?

- Ja

- Nein

23. Glauben Sie, dass ÄrztInnen mehr Anleitung für den Einsatz von Clozapin in der Behandlung von Menschen mit Schizophrenie brauchen?

- Ja

- Nein

24. Bitte beantworten Sie die folgenden Fragen. Antwortskala: ja / eher ja / eher nein / nein

- Fühlen Sie sich in der Identifikation einer therapieresistenten Schizophrenie sicher?

- Fühlen Sie sich hinsichtlich Ihres Wissens bezüglich der Empfehlungen/Leitlinien, wann Clozapin angeboten werden sollte, sicher?

- Fühlen Sie sich im Monitoring von PatientInnen mit Clozapin hinsichtlich ihres körperlichen und psychischen Befindens sicher?

- Fühlen Sie sich im Umgang mit Clozapin-assoziierten Nebenwirkungen sicher?

- Fühlen Sie sich im Absetzen von Clozapin (inkl. geplantes Absetzen und notfälliges, abruptes Absetzen) sicher?

- Glauben Sie, dass Clozapin effektiv gegen die Negatisymptomatik einer Schizophrenie wirken kann?

- Glauben Sie, dass Clozapin aggressives Verhalten bei PatientInnen mit Schizophrenie verringert?

- Glauben Sie, dass Clozapin die Mortalität durch Suizidversuche bei PatientInnen mit Schizophrenie verringert?

- Glauben Sie, dass Clozapin die kardiovaskuläre Mortalität verringert?

- Glauben Sie, dass Clozapin die Gesamtmortalität bei PatientInnen mit Schizophrenie verringert?

25. Verwenden sie Therapeutisches Drug Monitoring (TDM) allgemein in der Therapie mit Nicht-Clozapin-Antipsychotika?

- Ja

- Nein

- Nicht regelmäßig

26. Verwenden sie Therapeutisches Drug Monitoring (TDM) in der Therapie mit Clozapin?

- Ja

- Nein

- Nicht regelmäßig

27. Bitte ordnen Sie die u.g. möglichen Barrieren auf Seite der Verordnenden/Behandelnden für die Verwendung von Clozapin nach ihrer Bedeutung: oben = am bedeutendsten bis unten = am wenigsten bedeutend.

- Aufwändige Handhabung in Bezug auf Eindosierung, Nebenwirkungen und Labormonitoring-Anforderungen

- Fehlende Verwendung von partizipativer Entscheidungsfindung bei medikamentösen Therapieentscheidungen

- Fehlende Vertrautheit bzw. fehlende Schulung und Erfahrung der ÄrztInnen / PsychiaterInnen in der Verwendung von Clozapin

- Unsicherheiten bezüglich der Indikationsstellung

- Angst der Verordnenden / ÄrztInnen vor Clozapin-assoziierten Nebenwirkungen

- Erwartung, dass die Behandlung von den PatientInnen abgelehnt wird

28. Gibt es Ihrer Meinung nach weitere wichtige Barrieren auf Seite der Verordnenden/Behandelnden für die Verwendung von Clozapin, die in der vorangehenden Frage nicht genannt worden sind? Bitte geben Sie diese stichpunktartig an oder “Keine”, wenn es Ihrer Meinung nach keine weiteren mehr gibt.

29. Bitte ordnen Sie die u.g., möglichen Barrieren auf Seite der PatientInnen für die Verwendung von Clozapin nach ihrer Bedeutung: oben = am bedeutendsten bis unten = am wenigsten bedeutend.

- Monitoring-Anforderungen (z.B. regelmäßige Laborkontrollen)

- Angst der PatientInnen vor Clozapin-assoziierten Nebenwirkungen

- Belastung durch Clozapin-assoziierte Nebenwirkungen

- Fehlende Krankheitseinsicht

- Fehlendes Krankheitsgefühl bzw. fehlende Belastung durch die Symptome

30. Gibt es Ihrer Meinung nach weitere wichtige Barrieren auf Seite der PatientInnen für die Verwendung von Clozapin, die in der vorangehenden Frage nicht genannt worden sind? Bitte geben Sie diese stichpunktartig an oder “Keine”, wenn es Ihrer Meinung nach keine weiteren mehr gibt.

31. Wie sehr sind die folgenden Nebenwirkungen für Sie als BehandlerIn ein Hindernis für eine längerfristige Behandlung mit Clozapin? Bitte ordnen sie von oben = kein Hindernis bis unten= sehr großes Hindernis.

- Neutropenie

- Myokarditis

- Gewichtszunahme

- Obstipation

- Sedierung

- epileptische Anfälle

- Hypersalivation

- Akathisie

- Agranulozytose

32. Nach wie vielen erfolglosen Versuchen anderer Antipsychotika bieten Sie Clozapin an?

- 1 erfolgloser Versuch

- 2 erfolglose Versuche

- 3 erfolglose Versuche

- 4 erfolglose Versuche

- 5 erfolglose Versuche

- 6 erfolglose Versuche

- 7 erfolglose Versuche

- >7 erfolglose Versuche

- Ich verschreibe Clozapin nicht

33. Nach wie vielen erfolglosen Versuchen anderer Antipsychotika empfehlen die Leitlinien ihrer Einschätzung/ ihres Wissens nach einer Behandlung mit Clozapin?

- 1 erfolgloser Versuch

- 2 erfolglose Versuche

- 3 erfolglose Versuche

- 4 erfolglose Versuche

- 5 erfolglose Versuche

- 6 erfolglose Versuche

- 7 erfolglose Versuche

- >7 erfolglose Versuche

34. Wenden Sie bei Ihren PatientInnen vor Beginn einer Clozapin-Therapie üblicherweise eine Kombination anderer Antipsychotika an?

- Ja, einen Versuch mit der Kombination von Antipsychotika in therapeutischer Dosis

- Ja, zwei Versuche mit der Kombination von Antipsychotika in therapeutischer Dosis

- Ja, drei Versuche mit der Kombination von Antipsychotika in therapeutischer Dosis

- Nein, vor einer Kombinationsbehandlung unternehme ich einen Mono-Therapieversuch mit Clozapin

35. Wie lange kann man Ihrer Ansicht nach nach Feststellung der Therapieresistenz warten, bis man mit Clozapin beginnt?

- 1 Monat

- 3 Monate

- 6 Monate

- 12 Monate

- 48 Monate

- > 48 Monate

36. Bitte beantworten Sie die folgenden Fragen. Antwortskala: ja / eher ja / eher nein / nein

- Denken Sie, dass PatientInnen die Einnahme von Clozapin anderen Antipsychotika gegenüber vorziehen?

- Denken Sie, dass die Vorteile von Clozapin in der Behandlung der therapieresistenten Schizophrenie die potenziellen Nachteile überwiegen?

- Denken Sie, dass Clozapin zu selten verordnet wird?

37. Glauben Sie, dass Sie sich hinsichtlich der Initiierung einer Clozapinbehandlung und/oder dem Umgang mit Patienten, die Clozapin erhalten, sicherer fühlen würden, wenn Sie mehr Erfahrung im Umgang mit diesen PatientInnen hätten?

- Ja

- Nein

38. Was wäre Ihrer Meinung nach hilfreich, um die Clozapinverordnung und -verwendung in Ihrem Arbeitsbereich zu erleichtern?
